# GToTree: a user-friendly workflow for phylogenomics

**DOI:** 10.1093/bioinformatics/btz188

**Published:** 2019-03-13

**Authors:** Michael D Lee

**Affiliations:** Exobiology Branch, NASA Ames Research Center, Moffett Field, CA, USA

## Abstract

**Summary:**

Genome-level evolutionary inference (i.e. phylogenomics) is becoming an increasingly essential step in many biologists’ work. Accordingly, there are several tools available for the major steps in a phylogenomics workflow. But for the biologist whose main focus is not bioinformatics, much of the computational work required—such as accessing genomic data on large scales, integrating genomes from different file formats, performing required filtering, stitching different tools together etc.—can be prohibitive. Here I introduce GToTree, a command-line tool that can take any combination of fasta files, GenBank files and/or NCBI assembly accessions as input and outputs an alignment file, estimates of genome completeness and redundancy, and a phylogenomic tree based on a specified single-copy gene (SCG) set. Although GToTree can work with any custom hidden Markov Models (HMMs), also included are 13 newly generated SCG-set HMMs for different lineages and levels of resolution, built based on searches of ∼12 000 bacterial and archaeal high-quality genomes. GToTree aims to give more researchers the capability to make phylogenomic trees.

**Availability and implementation:**

GToTree is open-source and freely available for download from: github.com/AstrobioMike/GToTree. It is implemented primarily in bash with helper scripts written in python.

**Supplementary information:**

Supplementary data are available at *Bioinformatics* online.

## 1 Introduction

The number of sequenced genomes is increasing rapidly, largely through the recovery of metagenome-assembled genomes (e.g. Hug *et al.*, 2016; Parks *et al.*, 2017) and through the generation of single-cell amplified genomes (e.g. Berube *et al.*, 2018; Kashtan *et al.*, 2014). Phylogenomics (inferring genome-level evolutionary relationships) is becoming a fundamental step in many biologists’ work—such as in the characterization of newly recovered genomes, or in leveraging available reference genomes to guide evolutionary questions (Braakman *et al.*, 2017).

There are several tools available for the major steps in a typical phylogenomics workflow, and at least one analysis platform that incorporates a phylogenomics workflow amid a larger infrastructure (anvi’o; Eren *et al.*, 2015). But a complete workflow focused solely on phylogenomics, enabling greater efficiency and scalability, and with flexibility with regard to input formats, is lacking.

GToTree fills a void on three primary fronts: (i) it accepts as input any combination of fasta files, GenBank files and/or NCBI accessions—allowing integration of genomes from various sources and stages of analysis without any computational burden to the user; (ii) it enables the automation of required between-tool tasks such as filtering out hits by gene-length, filtering out genomes with too few hits to a specified target gene-set, and swapping genome identifiers so resulting trees and alignments can be explored more easily; and (iii) its scalability—GToTree can turn ∼1700 input genomes into a tree in 1 h on a standard laptop, and can optionally run many steps in parallel. This software gives more researchers the capability to create phylogenomic trees to aid in their work. At the time of publication, GToTree is primarily implemented in bash, but it will be converted to entirely python and be controlled by a more appropriate workflow language in the near future.

## 2 Description

### 2.1 Input

The required inputs to GToTree are (i) any combination of fasta files, GenBank files and/or NCBI assembly accessions, and (ii) an hidden Markov Model (HMM) file with the target genes. The HMM file can be custom or one of the 13 included HMM files covering varying breadths of diversity (discussed below). Optionally, the user can also provide a mapping file of specific input genome IDs with the labels they would like to have displayed in the final alignment and tree.

### 2.2 Processing

**Fig. 1.**
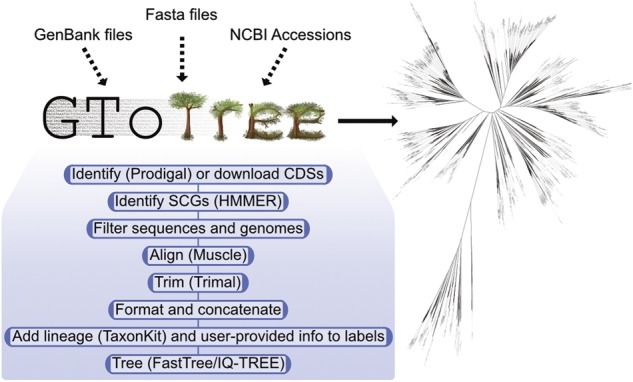
Overview of general workflow and an example Tree of Life made with GToTree encompassing ∼1700 genomes from NCBI’s RefSeq using a universal SCG-set (Hug *et al.*, 2016)

An overview of the GToTree workflow is presented in Figure 1 and detailed here:

1. Retrieve coding-sequences (CDSs) for input genomes, depending on the input source:
  - fasta files—identify CDSs with prodigal (Hyatt *et al.*, 2010)
  - GenBank files—extract CDSs if annotated, if not identify with prodigal (Hyatt *et al.*, 2010)
  - NCBI accession—download amino acid sequences of CDSs if annotated, if not, download the assembly and identify CDSs with prodigal (Hyatt *et al.*, 2010)
2. Identify target genes in all genomes with HMMER3 (Eddy, 2011) using pre-defined model cutoffs (–cut_ga)
  - by default, if a genome has more than one hit to a target gene, no gene will be contributed to the alignment for that target gene from that genome.
3. Report estimates of genome completeness/redundancy using the information from the HMM search (see Supplementary Note S1).
4. Filter out potentially spurious gene-hits based on length, and genomes based on fraction of target-genes detected.
5. Align each gene-set with Muscle (Edgar, 2004), perform automated trimming with Trimal (Gutíerrez *et al.*, 2009), and concatenate all.
6. Optionally add custom genome labels or lineages (for any that have taxids associated with them whether from NCBI accession or found in provided GenBank files; utilizes TaxonKit; Shen and Xiong, 2019).
7. Generate tree, currently supported are FastTree (Price *et al.*, 2010; note: FastTree does not enable incorporation of a specified root in tree generation) and IQ-TREE (Nguyen *et al.*, 2015; IQ-TREE does enable the incorporation of a specified root).

### 2.3 Outputs

The primary outputs from GToTree include the full alignment file (fasta), the tree file (newick), and tab-delimited summary tables with information on all genomes and individual ones for each genome input source. Additionally, outputs include report files on filtered or problematic genes/genomes.

### 2.4 CG-set generation

All 17, 929 Pfam (protein families; El-gebali *et al.*, 2019) HMM profiles from release 32.0 (accessed on December 2018) were downloaded from the Pfam ftp site (ftp://ftp.ebi.ac.uk/pub/databases/Pfam/). As Pfam-HMMs actually target-specific domains or protein regions, there are many unique Pfam entries that come from the same functional protein—e.g. Enolase_N (PF03952) and Enolase_C (PF00113). This is not ideal if using them to search for single-copy genes (SCGs) for purposes such as phylogenomics or completion/redundancy estimates). To ensure no two Pfam-HMMs from the same protein were contained in a SCG-set, only Pfams with HMMs that on average covered >50% of the underlying protein sequences that went into building that Pfam’s HMM were retained. This left 8924 Pfams.

To identify target SCGs, amino-acid CDSs of all ‘complete’ genomes with annotations in NCBI were downloaded for bacteria (*n* = 11, 405; accessed December 9, 2018) and archaea (*n* = 309; accessed December 15, 2018) (‘Complete’ is a specific classification of genome quality assigned by NCBI, see Supplementary Material Note S2.). All protein sequences were searched against the 8924 filtered Pfam-HMMs with ‘hmmsearch’ (HMMER v3.2.1; Eddy, 2011) with default settings other than specifying the ‘–cut_ga’ flag to utilize the gathering thresholds stored in the curated Pfam models. Reported protein hits for each individual Pfam were tallied for each individual genome (Supplementary Table S1; available at figshare.com/articles/Supp_Table_1/7562453). SCG-sets were generated for all Bacteria, all Archaea, and then for each bacterial phylum that held >99 genomes, and each proteobacterial class that had >99 genomes. For each of those taxonomic groups, Pfams that had exactly 1 hit in greater than or equal to 90% of the genomes of that group were retained as the SCG-set for that group. The counts for HMM hits for all genomes assayed are presented in Supplementary Table S2, and the code used to generate the bacterial SCG-set as an example is presented here: github.com/AstrobioMike/GToTree/wiki/SCG-sets.

## 3 Results

To exemplify GToTree, NCBI assembly accessions were downloaded for all RefSeq, complete, representative genomes (with the search query ‘“latest refseq”[filter] AND “complete genome”[filter] AND “representative genome”[filter] AND all[filter] NOT anomalous[filter]’ performed on Decemeber 20, 2018). This resulted in 1698 genomes spanning Archaea, Bacteria and Eukarya (please see Supplementary MaterialNote S3 on including Eukaryotes with GToTree). Using a SCG-set that spans all three domains (Hug *et al.*, 2016), runtime to create this tree (Fig. 1) was ∼60 min on a standard laptop (used was a late 2013 MacBook Pro). The tree was visualized by uploading the output newick file to the web-hosted Interactive Tree of Life (Letunic and Bork, 2016), all code to generate it and the results files come packaged with GToTree.

## Supplementary Material

btz188_Supplementary_Data
