# Supplementary material for: GToTree: a user-friendly workflow for phylogenomics
Source: Bioinformatics. 2019 Mar 13;35(20):4162–4. doi: 10.1093/bioinformatics/btz188 (PMC6792077; doi:10.1093/bioinformatics/btz188)
Supplement: btz188_Supplementary_Data [file btz188_supplementary_data.docx]

Supplemental text for GToTree: a user-friendly workflow for phylogenomics

**An important caveat on the idea of a “workflow for phylogenomics”**

Phylogenetics is an incredibly complicated and well-researched field, and things become even more complicated when working with many concatenated genes. GToTree is meant to be a relatively high-throughput, user-friendly, and reproducible workflow, something I believe is useful due to the high volumes of sequencing data and genomes we are often working with these days. But anything designed this way needs to sacrifice something in terms of flexibility and options. It is important that users new to this arena understand that many things impact the outcome of a phylogenetic/genomic analysis, particularly including the alignment algorithm used, and the model and program used for tree construction. At the time of publication, GToTree currently employs only one alignment tool, and two options for tree construction, though others will continue to be implemented, and following the wiki (github.com/AstrobioMike/GToTree/wiki) is a good way to stay updated. Users can also take the concatenated alignment output by GToTree and use that with many other tree construction tools. But it is important to keep in mind that phylogenetic analysis is complicated, and no one program or tool is an "absolute answer" – another way to think of it is with the old adage "all models are wrong".

**Note 1: Genome completeness and estimation**

Genome completeness is calculated as the number of target HMM genes identified divided by the total number of target HMM genes searched (* 100 to make it a percentage). So, if 100 single-copy genes (SCGs) were searched, and 95 were found, the estimated completeness would be 95%.

Genome redundancy is calculated as the number of copies greater than 1 that were identified for all target genes divided by the total number of target HMM genes searched (* 100 to make it a percentage). So, if 100 SCGs were searched, and 1 gene was detected in 2 copies, that would be 1 copy over the target: 1 / 100 * 100 = 1% estimated redundancy. If 1 gene was detected in 11 copies, that would be 10 over the target: 10 / 100 * 100 = 10% estimated redundancy. This would come out to the same estimate if 10 individual genes were detected in 2 copies each. This would still be 10 over the target: 10 / 100 * 100 = 10% estimated redundancy.

**Note 2: NCBI “complete” genomes**

NCBI assigns “levels” to genomes denoting their level of assembly. “Complete”, as noted on their website here (https://www.ncbi.nlm.nih.gov/assembly/help/), is defined as:

“*Complete genome - all chromosomes are gapless and have no runs of 10 or more ambiguous bases (Ns), there are no unplaced or unlocalized scaffolds, and all the expected chromosomes are present (i.e. the assembly is not noted as having partial genome representation). Plasmids and organelles may or may not be included in the assembly but if present then the sequences are gapless.*”

**Note 3: Are eukaryotic genomes appropriate for use with GToTree?**

If only using highly conserved ribosomal proteins, (like those in the Tree of Life example using the Hug et al. 2016 SCG-set), and/or if all genes are already identified (e.g. the input source is an NCBI accession with gene calls or a GenBank file with gene calls), then GToTree is suitable for working with Eukaryotes in addition to Bacteria and Archaea. If no gene-calls are available, then GToTree is likely not suitable for eukaryotic genomes as the only gene-caller currently implemented is prodigal.

**General overview**

Input files - any combination of fasta files, GenBank files, and/or NCBI assembly accessions

- fasta files - will identify coding sequences (CDSs) with [prodigal](https://github.com/hyattpd/Prodigal)
- GenBank files - will extract CDSs if they are annotated in the GenBank file, if not will identify them with [prodigal](https://github.com/hyattpd/Prodigal)
- NCBI assembly accessions - downloads NCBI assembly summary files, builds ftp links to download the appropriate assembly, attempts to download just the amino acid (AA) sequences of CDSs if annotations exist for it, if not will download the assembly in fasta format and identify CDSs with [prodigal](https://github.com/hyattpd/Prodigal) – examples of generating this accessions file from both the NCBI website and from the command line are shown in the [examples page](https://github.com/AstrobioMike/GToTree/wiki/example-usage#genomes)

Identify target genes

- GToTree then uses [HMMER3](http://hmmer.org/) to search each genome for the target genes specified the provided HMM file
  - 14 of these are provided with the software, listed on the [SCG-sets page](https://github.com/AstrobioMike/GToTree/wiki/SCG-sets)

Estimate genome completeness/redundancy

- using the information from the HMM search, reports estimates of % completeness and redundancy for each genome, also outputs a table of hits per target-gene per genome

Filter gene hits and genomes

- filter out genes based on length - get the median of all genes in that set, filter out those whose length is not within a certain range of the median length (20% by default)
- filter out genomes if they do not have hits to at least a certain fraction of the total genes searched (50% by default)

Add needed gap-sequences

- adds the appropriate-sized gap-sequences for target genes that are missing from genomes being retained in the analysis

Align, trim, concatenate

- align each gene set with [Muscle](https://www.drive5.com/muscle/)
- perform automated trimming with [Trimal](http://trimal.cgenomics.org/)
- concatenate all together into full alignment

Optionally add more informative headers - making things easily searchable in the resulting tree and alignment

- this can be done in two ways (one or the other, or both together)
  - use [TaxonKit](https://github.com/shenwei356/taxonkit) for those genomes that have taxids associated with them (whether from NCBI accessions or found in the provided GenBank files) to add lineage information to the genome labels
  - a two- or three-column tab-delimited mapping file can be provided with either the NCBI accession or input file name in column 1 (depending on input source), and the desired genome label in column 2, and/or text to append to the label in column 3 (not all input genomes need to be provided)

Tree

- tree with [FastTree](http://www.microbesonline.org/fasttree/) or [IQ-TREE](http://www.iqtree.org/)

Primary outputs include:

- the tree file and alignment file
- a genome summary table mapping all modified labels to original genome IDs, estimates of completion/redundancy, and any available taxonomy information
- a table showing number of hits per target-gene per genome
- reports on what, if anything, was filtered out at which steps
